# Unusual Quantum Transport Mechanisms in Silicon Nano-Devices

**DOI:** 10.3390/e21070676

**Published:** 2019-07-11

**Authors:** Giuseppe Carlo Tettamanzi

**Affiliations:** Institute of Photonics and Advanced Sensing and School of Physical Sciences, The University of Adelaide, Adelaide SA 5005, Australia; giuseppe.tettamanzi@adelaide.edu.au; Tel.: +61-(0)-883130248

**Keywords:** mesoscale and nanoscale physics, Complementary Metal Oxide Semiconductor (CMOS) technology, quantum transport

## Abstract

Silicon-based materials have been the leading platforms for the development of classical information science and are now one of the major contenders for future developments in the field of quantum information science. In this short review paper, while discussing only some examples, I will describe how silicon Complementary-Metal-Oxide-Semiconductor (CMOS) compatible materials have been able to provide platforms for the observation of some of the most unusual transport phenomena in condensed matter physics.

## 1. Introduction 

The analogy between the hydrogen atom and donor and acceptor dopants in semiconductors has been known for a very long time—see References [1,2] and references therein. However, mainly during the latter half of the past century, in experiments related to conventional semiconductor physics, dopant-atoms have been used as passive elements for several kinds of logic building blocks, for example, P-N junctions [1]. Under this original implementation method, many dopants were implanted in semiconductor materials to obtain a primitive control of their band gap and to create a barrier that could, in turn, be used to control the transition between the OFF state and the ON state (or vice versa) of a transistor made with these materials [1]. However, towards the end of the past century, limitations to this primitive approach to band gap engineering became apparent. Successively, many researchers around the world started to fabricate individual devices, which allowed much better control of the band gap via heterojunctions and other sorts of nanostructures [1,3]. A heterojunction is formed when two different semiconducting materials, typically with a small mismatch on the size of their lattice constants, are deposited one on top of the other. By doing so, it is possible to obtain a better control on the band gap of the materials, and consequently, to achieve fundamental advancements in the properties of optoelectronics devices [1,3,4]. While from their optical points of view, heterostructures fabricated via heterojunctions, such as quantum wells and superlattices, have already demonstrated the ability to achieve many of their electronic potentialities [3], zero-dimensional heterostructures (i.e., quantum dots), have also been introduced [3]. The latter potentially give rise to equivalent individual atoms in a semiconductor environment, and can be used as so-called artificial atoms [1,3,4,5]. In this context, there is a long list of materials, for example see References [6,7], that have been used for the observation of these exciting features of mesoscopic and “many-body” effects. In this short review paper, however, I will limit my discussion on some of the effects recently observed in devices fabricated with CMOS compatible materials [4,5].

## 2. Introduction to New Results

This review paper discusses how silicon CMOS compatible devices have recently revolutionized not only the fields of quantum information and quantum communications, e.g., see Ref. [4,5], but also those related to mesoscopic physics because of their demonstrated ability to compete with exotic, but not as yet commercially available materials as required for larger-scale applications, such as graphene [6] or carbon nanotubes [7]. Bearing in mind that silicon CMOS compatible devices remain the core platform for most of the traditional information processing infrastructures, this new development represents an impressive achievement [4,5]. 

To provide further details, this review paper aims at discussing how silicon CMOS compatible ultra-scaled devices can be used for the study of very elusive problems, for example the orbital Kondo effect [8,9], the Kondo-Fano effects [10,11] as well as the mechanism of errors in single electron pump (SEP) devices [12,13,14,15].

**Figure 1:**
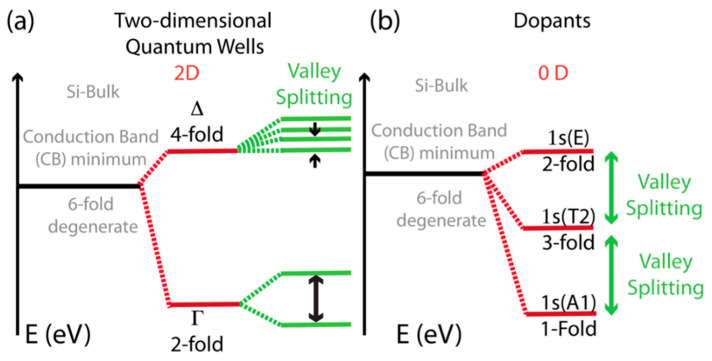
Schematics of the bottom of the conduction band (CB) for a few typical Silicon systems. The effects of confinement, of electrical effects and of structural atomic effects to the CB structure are included in (**a**) for two-dimensional Quantum Well and on (**b**) for an isolated dopant-atom impurity such as arsenic (As) or phosphorous (P), see also Reference [4]. Two-fold spin degeneracies are not included in this illustration [4].

One of the interesting aspects is that these silicon-based devices possess some peculiar and unique properties, if compared to the ones present in other semiconductor materials, due to the fact that, for silicon-based materials, the electron band gap exists in an indirect form [4,5]. These properties can be summarized by using the schematic picture of Figure 1 and by mentioning that electrons in silicon possess an extra degree of freedom, known as the “valley-orbital pseudo spin degree of freedom” [4,8,9,10,11,16,17], which occurrence is related to the fact that, for bulk silicon materials, the energies of electron forms in the conduction band (CB) are not minimized when the crystal momentum **k** is equal to 0 and are 6-fold degenerate at the minimum point [4,8,9,10,11,16,17]. The use of the term “pseudo-spin” in conjunction with the terms “valley” and “orbital” is somewhat controversial. Nevertheless, it is often used in many recent publications, see for example References [4,8,9,10,11,16,17] However, in this document, I have repetitively made use of this term in the context of silicon valley-orbital degrees of freedom. 

Consequently, the pseudo-spin can act as a good quantum number [18,19], and as Figure 1 schematically shows, the level degeneracy observed at the minimum of the CB can be partially or completely lifted by means of confinement effects see References [1,2,3,4] and references therein; hence, this degree of freedom can be utilized as a platform for novel quantum logic operations, see References [4,5] and references therein.

As an example, qubits based on the valley-orbital degree of freedom can be better isolated against the deleterious effects of charge noise as opposed to the ones based on more conventional degrees of freedom such as spin or charge [20]. Even though it is true that devices utilizing valley-orbit states are limited by the fact that deterministic control of the valley splitting is far from being routinely achieved [4,20]. In this review paper, I will show that, although the indirect bandgap properties of silicon were for a long time considered as a negative attribute of silicon materials, especially from the optoelectronic point of view, more recently these properties have demonstrated to be silicon material strengths, especially in view of the possible uses of silicon devices for quantum applications [4,5]. This is particularly true because of the numerous significant advancements made in the technologies used in the fabrication of silicon CMOS compatible devices [5]. In view of this, it is now possible to obtain extremely precise control of the quantum properties of electrons confined in silicon CMOS compatible structures even during fabrication [4,5] and it is also possible to engineer devices precise to a single atom level [21,22,23,24]. These new enhanced fabrication capabilities have translated into an improved ability for the control of all the quantum degrees of freedom (for example spin, charge, pseudo-spin) of electrons [8,9,10,11,12,13,14,15,16,17,20,21,22,23,24] and holes [25] in silicon nanostructures [4,5]. Finally, although there are still a few open questions in this area, for example see Reference [4], the achievements described above have translated into improved control of the electronic signature that can be observed in these devices. This, in turn, explains why these systems have also been used recently as platforms for the observation of some of the most remarkable effects in physics [4,8,9,10,11,12,13,14,15], in the same manner in which they were previously observed in other materials such as carbon nanotubes and graphene [6,7]. 

### 2.1. Special Properties of the Electrons in Silicon CMOS Compatible Devices

As already mentioned above and to be more specific regarding the characteristics of these silicon-based systems, it is important to note the fact that the energetic structure at the minimum of the conduction band of silicon devices fabricated on basic structures of quantum dots and single atom nanostructures can be quite different when compared to the energetic structures observed in bulk materials [1,2,3,4], see the schematic picture in Figure 1. 

**Figure 2:**
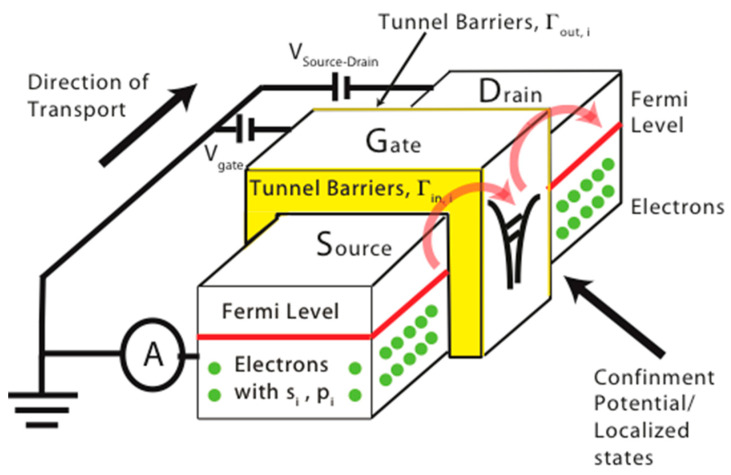
Schematic of a three-terminal device with one or more than one gate controlling the tunneling barrier from the source to the state, Γ_in,i_, and the tunneling barrier from state to the drain, Γ_out,i_. The leads (source/drain) represents an infinite reservoir of electrons with the all the possible spin (s_i_) and valley-orbital (p_i_) polarizations. The transport in the device can be controlled by applying a voltage to the V_gate_ terminal that controls the position of the quantum states in the confinement potential respectively to the Fermi level in the source and drain leads. In this configuration, the V_gate_ terminal can also control the transparency of the tunneling barriers Γ_in,i_ and Γ_out,__i_. The V_Source-Drain_ voltage at the drain terminal can control the polarity and the intensity of the current of electrons, while the source terminal is connected to a pico-ammeter. As the figure shows, all the elements of this circuit are connected to the same reference grounding.

This observation justifies the proven capability of silicon to host nano-devices, e.g., see schematic in Figure 2, which allow a good control of the spin degree of freedom of electrons, due to its weak spin-orbit coupling and to the existence of isotopes of silicon with zero nuclear spin [4]. Hence, by using silicon devices, it is possible to engineer a well-controlled quantum environment that can act as the ground for the observation of several exciting mesoscopic physics effects. These phenomena will be the focus of this review paper.

Several recent review papers and books, i.e., see References [4,5,26,27] and references therein, have described in detail many of the different approaches that can be used to fabricate these silicon devices. Consequently, my current review will simply refer to the relevant publication and, in instances where it is necessary to elaborate on a particular point, I will provide additional information covering fabrication techniques, without repeating what has already been reported in the relevant publications such as References [4,5,26,27].

In order to understand the peculiar behavior observable with silicon nanostructures [4,5,26,27], it is important to initially describe in detail the structure of the minima of the conduction band introduced above, see Ref. [1,2,3,4] and Figure 1.

As silicon has an indirect band gap [1,2] and because of its lattice symmetry, its conduction band minima has six degenerate minima (or valleys) at the point k = 0.85 k_0_ of energy vs. wave vector, **k**, diagram in the reciprocal space, as also schematically shown with the black lines in Figure 1, with k_0_ being the wave vector that defines the size of the unit cell in the reciprocal lattice space of the material [1,2]. This peculiar band structure, unlike other materials that have a direct band gap, e.g., gallium arsenide (GaAs), produces a situation in that electrons in silicon have an extra degree of freedom, which can be used for their quantum dynamical control; i.e., the valley-orbital pseudo-spin degree of freedom introduced above. As such electrons in silicon are said to be affected by multi-valley physics [4].

Furthermore, because, in these structures, most of the low temperature transport of electrons is located around the minimum of the conduction band, the multi-valley physical properties described in Figure 1 play a critical role for most of the mesoscopic low-temperature effects, as described in this review paper. Moreover, I have no desire to cover in the extensive amount of materials represented by the more than fifty years of research in the fields of silicon microelectronics [1,3] and silicon nano-electronics [4]. However, I would like to discuss some interesting and more recent progress in the field of silicon nano-electronics [4]. Moreover, another interesting aspect is that, as shown in Figure 1, both for donor impurities in silicon, such as arsenic or phosphorous, for two-dimensional (quantum wells) and for zero-dimensional hetero-structures (i.e., quantum dots), due to the severity of effects such as confinement, electric fields with strong gradients, lattice imperfections, and atomic-scale details at the interface between different sections of a device, most of the energy levels degeneracy at the minimum of the conduction bands can be lifted. For a Quantum Well (QW), the 6-fold valley degeneracy is broken by the large in-plane tensile strain to a 2-fold degenerate Γ levels that are below 4-fold degenerate Δ levels [4]. These degeneracies can in turn be broken by all other atomic effects that go under the name of valley-splitting see Figure 1a). For isolated dopant-atoms in the silicon lattice, i.e., single atom transistors [8,9,10,11,12,13,17,21,22,23,24], in the most ideal situation, the final configuration gives a 1-fold degenerate 1s state (A1), below a 3-fold degenerate 1s and another 2-fold 1s state, as shown in Figure 1b), while other intermediate situations are possible [8,9]. As an example, the lower energetic valley-orbital states are fundamental for the observation of the Kondo and the Kondo-Fano effects described in the section below. These properties are also a fundamental ingredient for the successful operation at high frequency (≥GHz) of silicon based quantum electrons pumps [12,13,14,15]. 

The lifting of the valley-degeneracy of the electrons in quantum dot or quantum well devices in silicon can have multiple consequences; first it can allow the observation of novel quantum effects [8,9,10,11,12,13,14,15]. Furthermore, the study of this novel degree of freedom has demonstrated that this valley (pseudo-spin) degree of freedom can also act as a good quantum number [18,28] for electrons, and as such, it could be used for their coherent control in the implementation of quantum gates [4,20,28]. 

It is also important to clarify that, when electrons are confined in isolated dopant atoms or QD nanostructures an orbital order for the energy levels of the states also appears [4]. This orbital order in the hierarchy of the states can co-exist with the hierarchy of the distribution imposed by the physics of valleys [4]. Sometimes valley and orbital effects can lead to very distinguishable consequences in the structure of the energy levels [4], however, because these effects can typically be influenced in many ways by the microscopic structures of these devices, the most common way for valley/orbital effects to manifest themselves in silicon nano-structures is in a mixed configuration [4]. This explains why, for most silicon nanostructures, the term “valley-orbit degree of freedom” is often in use as opposed to the terms “valley degree of freedom” or “orbital degree of freedom” [20,28]. It is also interesting to briefly mention that the signature of these complex valley-orbit effects imposes selection rules that lead to dramatic alterations of the observed electronic effects [28].

### 2.2. Kondo Effects in Silicon Nanostructures

The three-terminal nano-device [4,5,8,9,10,11,12,16,17], illustrated in Figure 2 is a good example of the most simplified geometry that can be used to observe the quantum effects in silicon, when operating at sufficiently low temperatures. 

As shown in Figure 2, in this type of device, two terminals are used for the source and the drain leads, which therefore dictate the direction of transport according to the polarity of the bias voltage applied on the V_Source-Drain_. The third terminal is typically used to apply a voltage to the V_Gate_ and in turn, to control the transition of the device between the ON state and the OFF state at room temperature [1,29,30] and the position of the quantum level that is present in this system at low temperatures when Coulomb effects are observable [4,29,30]. For geometries, like the one shown below in Figure 2, the V_Gate_ can in principle also control the transparency of the tunneling barriers Γ_in,i_ and Γ_out,i_. While only more complicated structures, for example see the double gate structure, as discussed later on in this review paper, may offer the possibility for an independent control of the position of the quantum states and of the transparency of Γ_in,i_ and of Γ_out,i_, even a simple structure like the one described in Figure 2, it is possible to observe many-body effects at sufficient low-temperatures [8,9,10,11,12]. In this review paper, I will often use this geometry as a platform for the description of the interesting effects that can be observed in silicon nano-devices, since the said platform has already demonstrated the capability to offer access to the quantum systems, as illustrated in Figure 1.

**Figure 3:**
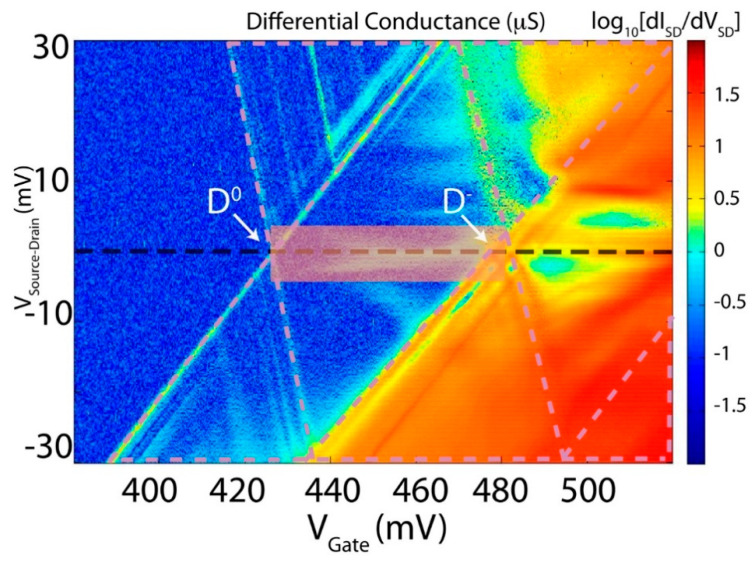
Example of Coulomb Blockade data similar to the ones in [7,8] and taken at 290 mK in a three-terminal device as the one schematically described in Figure 2. As an example, according to the orthodox Coulomb blockade theory, transport should arise only in the region delimited by the triangles contained within the pink dashed lines. Any transport signature outside these regions is linked to higher order effects [4,8,9]. An example of the latter is the signal present in the low bias, i.e., |V_Source-Drain_| < 5 mV, region between the one-electron (i.e., D^0^) and the two-electron (D^−^) charge states [4]. This signal, also outlined with the rectangular transparent area, is linked to the observation of spin and orbital Kondo fluctuations. The low bias region after the two-electron D^−^ is most likely linked to the occurrence of the Kondo based on fluctuation of integer degree of freedom [34].

The most typical first order regimes of transport, i.e., non-involving virtual states [4,8,9], which can be observed in three terminal nano-devices at low temperatures are the sequential and the coherent regimes of transport [4]. A typical Coulomb Blockade differential conductance (G = ${\mathrm{dI}}_{SD}/{\mathrm{dV}}_{SD}$) signature is shown inside the areas delimitated by the pink dashed lines in Figure 3. In this typical Coulomb Blockade situation, only first order transport is expected to arise, and only within those regions limited by the pink lines, while everywhere else no transport should be allowed [4]. 

However, from Figure 3, it is also clear that, for these systems, it can be affected by many higher order body effects. As such transport can arise in regions represented by the stability diagram in Figure, where it is not expected to, if only first order results are taken into consideration [4]. In fact, in this section, we are mainly interested in discussing in more detail some of these unexpected effects. These involve coherent and incoherent second order charge transitions, such as the ones related to the apparent violation of Heisenberg uncertainty principle and indeed can arise outside the conventional Coulomb Blockade picture. The Kondo, see References [8,9] and references therein, and the co-tunneling, see References [4,31], regimes of transport become observable when the temperature of the system is below a certain critical temperature (e.g., the Kondo Temperature) and when quantum fluctuations in the spin or in the pseudo-spin degrees of freedom, as opposed to temperature ones, become the leading fluctuation effects [4,8,9,10,11,32,33,34].

#### More about These Kondo Effects

**Figure 4:**
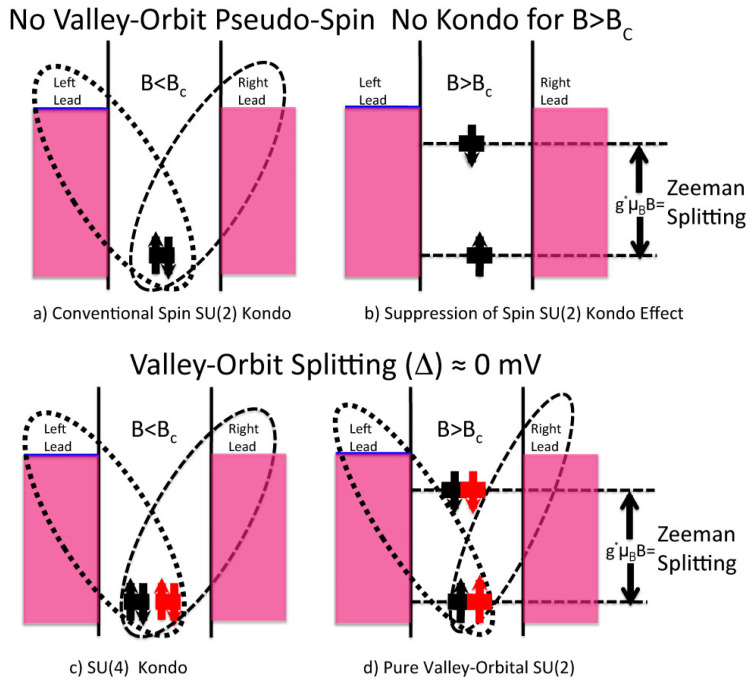
For conventional Quantum Dot Systems where only the fluctuations of spin degree of freedom are available [32], the Kondo effect is suppressed for sufficiently high magnetic fields (B_C_) because of the Zeeman splitting makes energetically impossible for Kondo fluctuations to arise. Opposite to this, when observed in an opportunely tuned silicon system, the Kondo Effect can arise as the combined action of the fluctuations of the spin and of the pseudo-spin (Valley-Orbit) degrees of freedom. As the pseudo-spin is typically only lightly affected by the magnetic field, a pure Orbital version [7,8,24,32] of the Kondo effect survive even for B > B_C_.

In this section, by observing the schematic of Figure 4, I will describe the main features that can be associated with the different versions of these Kondo effects. 

In its most conventional manifestation, the Kondo regime of transport is linked to the availability of the electronic spin degree of freedom only [32]. In this situation, this effect is suppressed as soon as a sufficiently high magnetic field is applied to the system. This is schematically illustrated in the diagram of Figure 4a, which shows that when a dual spin degenerate level is available and if there is no external magnetic field applied, then quantum fluctuation (schematically described in Figure 4 with the dashed loops when observable in the current signature) can be effective in allowing transport in the Coulomb Blockade forbidden regions. Figure 4b also illustrates that, when a sufficient external magnetic field is in place and if the two spin levels are sufficiently separated energetically due to Zeeman splitting, fluctuation effects cannot be sufficiently efficient in generating an observable current [8,9,10,11,32,33], i.e., the Kondo effect is indeed suppressed. This effect is also observable in the experiments [32].

The simple picture introduced in the section above and in Figure 4a,b can become more complicated for systems for which the valley orbit pseudo-spin degree of freedom is also available for Kondo fluctuations, in the same manner as silicon systems, as illustrated in Figure 1 [8,9,10,11].

For the latter, a very unusual variety of Kondo, i.e., pure orbital Kondo, can also be observed [8,9,10,19,33], because valley-orbital pseudo spin is only lightly affected by magnetic field splitting. The occurrence of pure orbital pseudo-spin [19], i.e., not involving fluctuations of the spin of the electrons, Kondo effect in silicon nanostructures, is discussed here below:

(a) For conventional semiconductors, the Kondo effect has only been observed in relation to interactions between the spin of electrons confined within the localized state and the ones of the surrounding free electrons at sufficiently low temperatures (T’s), i.e., for Temperature < Kondo temperature (T_Kondo_), see Reference [32]. This situation is illustrated in Figure 4a, and as shown in Figure 4b, in this situation, the spin-Kondo effect is suppressed when a sufficiently high magnetic field is applied to the system because the Zeeman splitting between the spin up and the spin down of the electrons makes energetically impossible for spin fluctuations to generate virtual states that would open the Kondo transport channel [32]. This situation goes also under the name of conventional SU(2) Spin Kondo effect.
(b) In materials were the pseudo-spin degree of freedom is also available, however, as in the area in Figure 3 outlined by the rectangular shaded shape and as illustrated in Figure 4c, the Kondo effect is somehow different to the one shown in Figure 4a and as described in the above sections. A different situation from the one above has recently been observed and is evident both from the experimental and from the theoretical points of view in silicon CMOS three terminal devices [8,9]. The extension of the Kondo effect to valley-orbital degree of freedom is clearly illustrated in Figure 4c by introducing different colors (black and red) for the two-different valley-orbital levels involved in the effect, i.e., the two lowest states, as shown as degenerate in Figure 4c,d. Consequently, the Kondo effect observed in silicon nanostructures is a more sophisticated phenomenon that goes under the name of SU(4) Kondo effect [4,8,9].

What makes these latter results even more interesting is the fact that a previously unobserved Kondo ground state symmetry crossover can be studied in these systems [8], because, as from one side it is possible to saturate the spin degree of freedom by mean of Zeeman splitting, as it is shown in Figure 4b in conventional Kondo systems, and from another side it is possible to be in a situation where the pseudo-spin fluctuations are still in place even if under the effect of a sufficiently high magnetic field, B > B_c_, with B_c_ being a certain critical field [8]. As shown in Figure 4d, in this situation, it is possible to saturate only the spin degree of freedom, but also to observe a Kondo effect originating only from the valley-orbital quantum fluctuations, the so-called “pure” SU(2) orbital Kondo effect [8,9,19]. These results imply pure quantum screening of the orbital degree of freedom [8].

**Figure 5:**
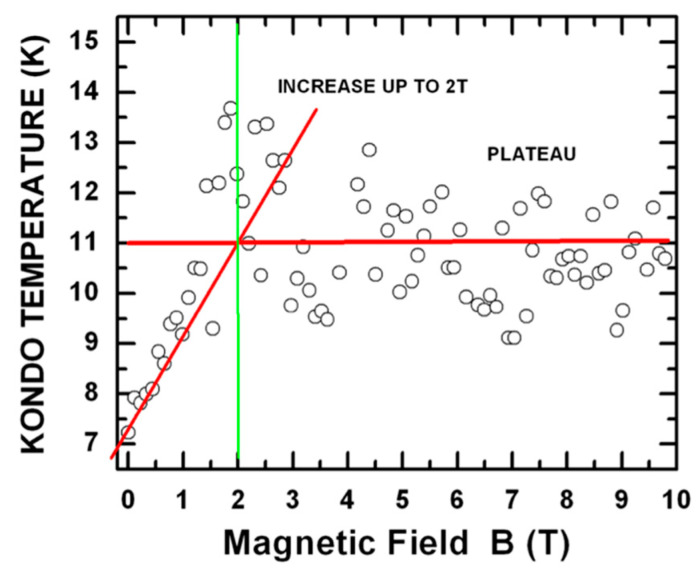
Universal law of the smooth transition between two different versions of the Kondo Effect (because observable in systems very different one from of each other [8,35]) under the effect of an external magnetic field (i.e., from an SU(4) Kondo to an SU(2) Kondo effect). Even if this data does present some scattering, nevertheless, an initial linear behavior of the order parameter (Kondo Temperature, T_C_) can be observed between 0 Tesla and 2 Tesla. This is most likely followed by a constant value of T_C_ for any B > 2 Tesla.

Due to the difficulty of accessing these regimes, the observation of these novel effects represents not only an important milestone for quantum many-body physics, but has also opened-up new pathways for silicon quantum electronics such as, for example, valley-orbital quantum bits [4,20]. Of course, these many-body effects cannot be directly used for the implementation of novel quantum information schemes, but their observation is very important, because it has permitted the study and the characterization, in a coherent fashion, of quantum states based on these pseudo-spin degrees of freedom that could, in the future, become the base for novel quantum schemes [20]. What makes the transition between the different versions of the Kondo effects, as illustrated in Figure 4c,d, ever more interesting is that this transition represents a universal phase transition equivalent to the ones observed in other systems having the same symmetry in the quantum degrees of freedom, e.g., some nuclear systems [35]. As shown under Figure 5, in this latter situation, a survival of a T_K_ ≠ 0 and a constant evolution of this T_K_ ≠ 0 are observed even for B > B_c_. While for conventional SU(2) Kondo for B > B_c_ T_K_ is = 0.

In conclusion, as also shown in Figure 5, by looking at the evolution of the order parameter of systems that have both spin and pseudo spin degrees of freedom (i.e., T_K_ in the case of our systems), it is possible to observe a unique universal law for the smooth transition between two different versions of the Kondo effect under the influence of an external magnetic field. These T_K_’s can be extracted by fitting the curves that describe how the current signature evolves at different temperatures [8]. Even if the data in Figure 5 does present some scattering, nevertheless, the experiment opens up an unique window to the characterization of universal physical behaviors that are expected to be observable in different systems [8,9,35]. 

### 2.3. Kondo-Fano Effects in Silicon Nanostructures

Another interesting aspect of the Kondo effect is that it provides the opportunity, when the quantum channel of transport is coherent, to observe phenomena that have for a long time been confined to only the optical side of the physical experiments. In this context, silicon nano-systems like the ones discussed in the previous sections, have recently been able to demonstrate [10,11] that they can manifest the Fano effect, which consists in the interference of a discrete coherent channel with a continuum and which gives rise to characteristically asymmetric peaks in the response [10,11,36]. Another way to describe this effect is by mentioning that specially shaped asymmetrical peaks, in this case current peaks, will appear every time with a path of rapid phase variations with almost constant phase interferences [10,11,36]. In a geometry like the one illustrated in Figure 2, the interference pathway can arise when more than one channel of conduction becomes available between the source and the drain terminals. This multi-channel configuration, in conjunction with the Aharonov-Bohm effect [37], allows the observation of the version of the effect that goes under the name of Fano-Kondo effect [10,11]. In the example discussed in References [10,11], the constant continuum is provided by a sequentially tunneling channel, while the coherent channel is provided by the Kondo effect due to correlated effect in the Coulomb Blockade regime as described in the previous section of this paper. Each of these two channels is linked to the transport via an atomic confinement potential located somewhere in the channel of the three-terminal device. A slave-boson mean-field approximation within the scattering matrix formalism was used to ensure that the data observed in Reference [10] was correctly interpreted within the framework of the Fano-Kondo effect [11].

### 2.4. Charge Pumping Effects in Silicon Nanostructures: Single Electron Pumps

**Figure 6:**
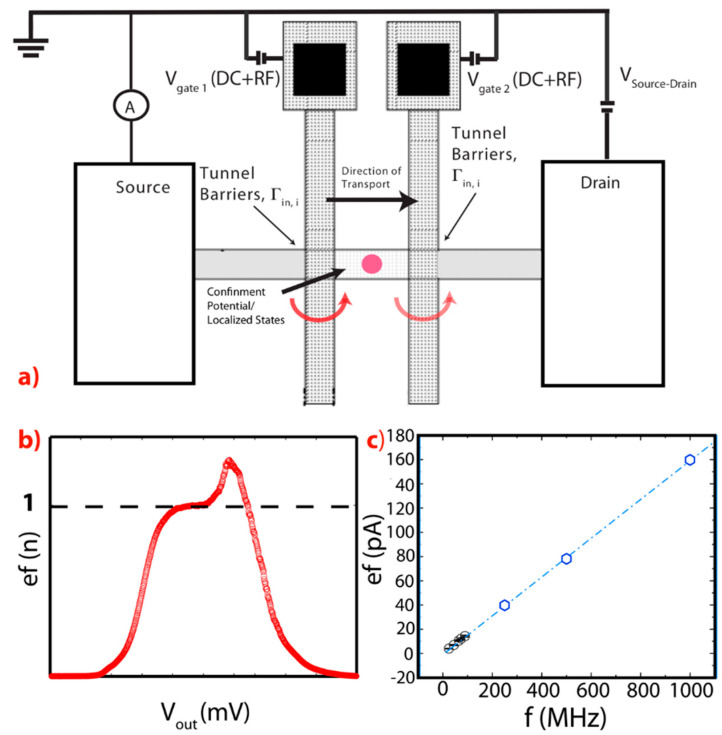
(**a**) Schematic of a typical measurement setup for a Single-Electron Pump [11,12,13,14]. (**b**) Characteristic *nef* response for *n* = 1 for a Single-Electron Pump [12]. (**c**) Characteristic *ef* response at different f (from a few MHz to 1000 MHz) for a Single-electron Pump [12].

In 1983, D.J. Thouless published a seminal paper [38] predicting that a periodic perturbation of the confinement potential for electrons in a nanostructure would, in principle, allow the generation of topologically protected band levels. These levels could lead to energetic regions where the transport of electrons from the source to the drain could be precisely controlled, even when the voltage between the source and the drain is zero, which statement is an apparent violation of one of the fundamental principle of electronics, Ohm’s law. By taking advantage of Coulomb Blockade phenomena’s and of quantum tunneling effects [4,5], this concept was expected to enable the generation of ultra-accurate currents [12,13,14,15]. See Figure 6a for an illustration of a typical setup that can be used for the kind of measurements described in this section [12,13,14,15].

The quantification of the currents generated under the above-mentioned technique, which goes under the name of quantized pumping, is based on the formula I_Source-Drain_ = n*f*e, see Figure 6b,c, where *e* is the elementary charge (1.6021766208 × 10^−19^C), *n* is a positive integer indicating the number of transported electrons for each period or cycle, and *f* is the frequency of perturbation of the confinement potential, with *f* = 1/*τ* and *τ* being the period. This technique allows the implementation of high performant clocked single-electron sources, i.e., generating sufficiently high currents with sub-parts per million (sub-ppm) uncertainties [12,13,14,15]; thanks to shot noise being naturally suppressed in these experiments [39], while adverse temperature and flicker noise effects can be substantially suppressed [12,13,14,15]. The basic idea of these experiments aims at the generation of a current flowing through a quantum dot/single atom impurity without applying a voltage between the leads, but by specifically varying potential at one or more gates. Furthermore, as Figure 6a shows, the schematic of the most common devices that are used for charge pumping experiments are slightly more elaborated, if compared to the one discussed in Figure 2, see also the many device geometries described in Reference [5].

**Figure 7:**
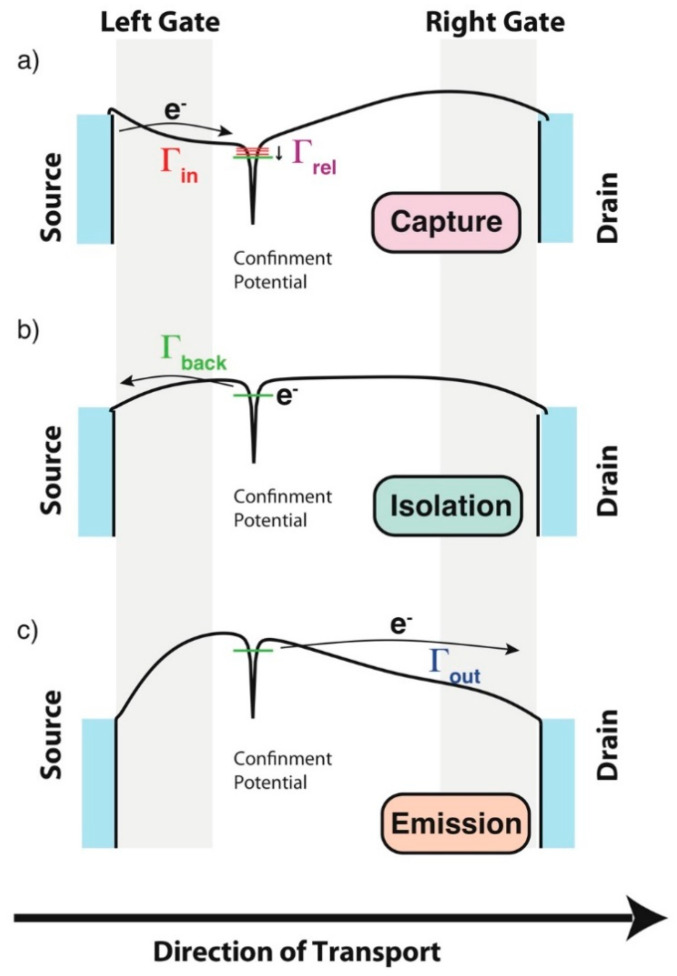
Ideal pumping cycle for a single atom pump [12,13] showing a schematic description for the different steps; (**a**) the capture section of the cycle, (**b**) the isolation, and (**c**) the emission.

In Figure 6a, it is shown that at least two terminals are needed for an independent control of the two gates and, therefore, of an independent control of the in-to state and the out-from state tunnel barriers (i.e., Γ_in_ and Γ_out_). This is different from the geometry schematically described in Figure 2 where only one terminal was used for the control of both these tunnel barriers. One of the most important aspects of this research is the fact that by operating the voltage gates in an opportune way, it is possible to obtain the appropriate sequential time-evolution of the voltages applied to the two gates, and therefore it is possible to generate an opportune time-evolution of the transparency of the tunnel barriers (i.e., Γ_in,i_ and Γ_out,i_) that allows the transport of exactly *n* electrons between the source and the drain each cycle [40]. The number of cycles per seconds (=*f*) will then determine the intensity of the current according to the law *I*_Source-Drain_ = n*f*e. This description of the quantum pumping current clarifies why these experiments where the current is made by controlling electrons one-by-one are expected to generate precise/accurate currents. A sequence schematically describing the different sections of an ideal pumping cycle for a single atom pump [12,13] is set out under Figure 7, describing (a) the capture section of the cycle, (b) the isolation section, and (c) the emission section [13].

Finally, if ideal quantum pumping transport is achieved, it is possible to obtain an ultra-fast and accurate circulation of an electrical charge (i.e., electrons) from the source to the drain [12,13,14,15,40]. The technical details on the manner in which these experiments can be performed is beyond the scope of my present review, see for example, the experiment descriptions in References [12,14,15]

I would like to conclude this review paper by describing in more detail the physics of the errors/non-ideal behaviors that can be observed in quantum pumps and I would like to discuss how these errors are, in particular, linked to silicon valley-orbital effects. Such errors can be seen as detrimental effects as they can cause the degradation of the currents measured in these charge pumping experiments. Interestingly, those experiments that were originally aimed at obtaining an accurate quantum definition of the ampere [12,13,14,15], more recently have opened up new directions in the fields of quantum information science [41,42,43] and classical signal processing [44].

### 2.5. Errors during the Operations of Single Electron Pumps

To understand the mechanisms of operations of an ideal single electron pump, it is important to remind ourselves that the main result, which is required when these devices are in operation is to obtain the most accurate possible control of electrons during each one of the fast cycles that are used for the circulation of electrons from the source to the drain via the localized state. Hence, the mainstay of these experiments relies on the ability to obtain a synchronized control of all the tunneling rates; the ones that control the movement of electrons from the source to the ones of the levels of the localized state (i.e., Γ_in,i_) and the ones related to the movement of electrons from the localized state to the drain (i.e., Γ_out,i_) [12,13]. Of course, the different relaxation rates internal to the localized state will also play a fundamental role in these experiments [12,13,15,25,45,46,47,48,49,50,51,52]. Here, it is important to mention that these experiments are mostly performed with source-drain bias at 0 V or in a region for which changes in the values of the source-drain bias are non-influent [12,13,14,15], i.e., the situation has given rise to the assumption that these pumping experiments represent a violation of an ideal definition of Ohm’s law [38].

The central idea of these experiments is to obtain optimal dynamical control of the different tunnel barriers that control the movement of electrons from source to drain. In turn, this is expected to lead to an optimization in the transport properties even when high frequencies, f, of operations are in use [12,13,14,15]. The conventional model describing the ideal operations of single-electron pumps, based on quantum dot (QD) confinement site, is often described as the Decay-Cascade model [46]. In this model, the pumping cycles can be schematically described with these three cyclical steps: (a) Capture from the source which for a QD is an easy occurrence because the dot is typically semi-open in the first section of the cycle [15,46]; (b) isolation into the localized state of *n* electrons; and (c) emission to the drain of *n* electrons, see also Figure 7 for a description of a similar cycle valid for a single atom pump [12,13]. If the ideal picture described above is followed during experiments, then the system will generate a current that is exactly equal to n*f*e.

However, I would mention that something could go wrong during each of the steps described above and the quantum pump could lose control of a certain number of electrons for each cycle. In turn, this can be the cause of degradation in the precision/accuracy of the current produced by the single-electron pump, and therefore this can lead to the generation of errors. As set out below, I have endeavored, based on the simplified schematic of Figure 7, to provide a simplified explanation to the most important mechanisms of errors that can be observed in these systems:

(a) The first mechanism of error is the one that becomes important when the temperature of operation (T) of the single electron pump is energetically comparable to the charging energy (E_C_) of the confined states, i.e., E_C_ ≈ K_B_T, with the charging energy being the energy that the system must pay to increase by one electron the number of electrons in the localized state between the two gates, i.e., from N to N + 1. These thermal effects could lead to losing control of many electrons in each step of the cycle described in Figure 7. The probability of the occurrence of this type of errors is linked to the formula $e^{-\frac{E_{C}}{K_{B}T}}$ Hence E_C_ >> K_B_T means that this probability is almost zero. As K_B_T~24 meV for T = 300 K and ~0.24 meV for T = 3 K, the typical E_C_~30–50 meV observed in single impurity/atom systems makes that these are immune to temperature errors even when they are operated above the liquid Helium temperatures (≥4.2 K). This explains why single-atom based single electron pumps (SAP’s) are extremely advantageous, as they do not need to be kept at ultra-low temperatures (<1 K) to operate in an environment completely immune from detrimental temperature effects/errors [12,13]. QD electron pumps are often limited in this sense as E_C_ for these systems is often limited to less than a few meV and as such require some complicated sub-kelvin temperature of operations to demonstrate their best performances [14]. However, as soon as the E_C_ of a QD increase to a value like the ones observed naturally in isolated single atom systems, for example by electrostatic confinements [14,47], these errors can also be suppressed in QDs electron pumps operating at temperatures ~4.2 K [14,15].
(b) Another mechanism of errors that can affect single electron pumps, when they operate slightly above the 100 MHz frequencies of excitation, is the one related to non-adiabatic effects [45,48]. These errors are related to the poor efficiency in the achievement of the second step of the pumping cycle, as described in Figure 7b, i.e., the isolation step. This poor efficiency can be observed when the rates that control the back-tunneling of the electrons from the localized state back to the source, Γ_back_’s, allow the escape of the electrons to the source before the full isolation or before the emission to the drain [46,47,48]. This kind of error can particularly affect QD electron pumps as, for these systems, electrons are strongly affected when fast perturbations are exciting the system. At high frequencies of operations, these excitations, i.e., non-adiabatic excitations [48], can lead to the delocalization of electrons between the ground-state and the excited states and as for QDs the rates that govern the tunneling between the excites state and the source/drain leads are fast, if compared to the ones between the ground state and the source/drain leads, hence, when electrons are delocalized, their probability of non-completion of the isolation step is much higher than normal [40,48]. Ultimately, this could lead to errors since it means that electrons will not be emitted to the drain and will not complete their cycle [48].

The detrimental mechanisms described above are much less likely to happen in SAPs as for these systems the shape of confinement potential is not as affected by thermal or non-adiabatic effects as much as it is in the case of QDs pumps.

Consequently, even when they are operating at 4.2 K and at GHz frequencies of excitation, non-adiabatic effects are not as efficient in causing errors in SAPs, as opposed to QDs pumps [12,13]. Furthermore, for SAPs the relaxation rates (Γ_relaxation_, see also schematic in Figure 7a) controlling the relaxation of the electrons from excited states to the ground states are considerably faster [24] when compared to the equivalent ones observed in QD pumps [48]. Here, it is very important to emphasize that the fast relaxation rate effects observed naturally in SAPs are directly linked to the energy spectrum that the multi-valley physics imposes on these silicon systems [12,13,24]. Thus, for SAPs, the isolation step of the pumping cycle can always be reached efficiently. This also leads to a different way to operate these quantum pumps, based on the initial capture via excited state and sub-sequent fast relaxation to a well-isolated ground state [12,13]. As such, it is important to outline that the high performances observed in SAP’s are linked to the indirect band gap properties characteristic of silicon materials. It is also important to associate the non-adiabatic effects observed for *f* = 1/*τ* between 100 MHz [48] and a few GHz [45] to a recent set of results hinting to the ability of studying the ultra-fast coherent dynamics of electrons in highly reproducible silicon CMOS compatible devices [45].

(c) The alternative way to operate a SAP described above can be relatively error-free, unless these systems are excited to frequencies considerably higher than the GHz ones [12,13]. Consequently, the discussion above opens the way to the description of another kind of errors that could arise in QD or in single atom pumps [13] when electrons reach the confinement potential via an excited state and not the ground state. If the *f* = 1/*τ* approaches the values of Γ_relaxation_ described in Figure 7, see also Reference [13]. In this situation, the electrons do not have sufficient time to relax to the ground state and the completion of the isolation step is compromised. The picture above can also be used to understand the causes of errors and of the degradation of the precision/accuracy of the measured currents in SAP’s [13].

Other complicated versions of the kind of errors described under sections (b) and (c) above have been observed in other systems [50,51,52] and are often linked to the fact that more than one confinement site is playing a role in the control of electrons [50,51,52]. It is important to remind that multi-valley physics effects play an important role in the dynamical evolution of all these errors, see for example Reference [13].

(d) Lastly, I would like to briefly discuss another possible mechanism of error that can cause the degradation of the current and which has recently been observed in a silicon QD system [15]. For a system where a QD pump is operating at ultra-fast frequencies of excitations (up to 3.55 GHz), it has been shown that the ideal behavior of the pump can sometimes be affected by errors that appear when an impurity-trap state can compete with the main QD in the capture and in the emission of the electrons [15]. Note that the eventual presence of impurity-trap states in the gate stack of silicon devices is a well-known fact [1,4,45]. This novel frequency dependent mechanism [15], has not been completely explained, and it is a reminder that for silicon CMOS compatible technology, although extremely controlled and reliable [4,5], it is still possible to observe some unexpected behaviors. It is however comforting to note that the hybrid dot-impurity systems, such as the one discussed in this Refs. [15,45], have recently been able to provide record high performances in term of frequency and accuracy of operations [15,50,51,52,53], but have also opened up the way to the use of the quantum pumping technology for novel quantum information schemes [45].

## 3. Conclusions

In this short review paper, I have discussed some of the most unusual quantum transport effects that have been recently observed in silicon CMOS compatible devices. If from one side valley-orbital effects can be linked to the novel observation of effects such as for example the SU(4) Kondo [8] and the Fano-Kondo ones [10], from another side single-atom transistors and other silicon devices [4,12,13,14,15,46,48], because of their immunity to thermal and non-adiabatic detrimental effects, have demonstrated the ability to provide a unique environment for the ultra-fast control of electrons [12,13,14,15,46,48]. It is also very interesting to observe that even in the quantum pumping regime, the special valley-orbit energetic structure of silicon nano-materials plays a fundamental role in opening the way to high-performant devices. This is a special outcome as the origin of these powerful effects, i.e., the indirect band gap properties observed for silicon, was in the past considered one of main limitation of these materials [1,2,3,4].
